# The effect of aging and caloric restriction on murine CD8+ T cell chemokine receptor gene expression

**DOI:** 10.1186/1742-4933-4-8

**Published:** 2007-11-14

**Authors:** Raymond Yung, RuRan Mo, Annabelle Grolleau-Julius, Mark Hoeltzel

**Affiliations:** 1 Department of Internal Medicine, University of Michigan, Ann Arbor, MI, USA; 2 Geriatrics Research, Education and Clinical Center, Ann Arbor Veteran Affairs Medical Center, Ann Arbor, MI, USA; 3 Department of Pediatrics, University of Michigan, Ann Arbor, MI, USA

## Abstract

**Background:**

The mechanism explaining the increased disease susceptibility in aging is not well understood. CD8+ T cells are crucial in anti-viral and anti-tumor responses. Although the chemokine system plays a critical role in CD8+ T cell function, very little is known about the relationship between aging and the T cell chemokine system.

**Results:**

In this study we have examined the effect of aging on murine CD8+ T cell chemokine receptor gene expression. Freshly isolated splenic CD8+ T cells from old C57BL/6 mice were found to have higher CCR1, CCR2, CCR4, CCR5 and CXCR5, and lower CCR7 gene expression compared to their younger cohort. Anti-CD3/anti-CD28 stimulation elicited a similar robust chemokine receptor response from young and old CD8+ T cells. Western blot analyses confirmed elevated protein level of CCR4 and CCR5 in aged CD8+ T cells. Increases in T cell CCR1 and CCR5 expression also correlate to increased *in vitro *chemotaxis response to macrophage-inflammatory protein-1 α(MIP-1α). Finally, caloric restriction selectively prevents the loss of CD8+ T cell CCR7 gene expression in aging to the level that is seen in young CD8+ T cells.

**Conclusion:**

These findings are consistent with the notion that aging exists in a state of low grade pro-inflammatory environment. In addition, our results provide a potential mechanism for the reported aging-associated impaired T cell lymphoid homing and allograft response, and reduced survival in sepsis.

## Background

The aging-associated susceptibility to infection and cancer is often attributed to the decline in immune response in the elderly [1]. Impaired T cell proliferation, receptor signaling, interleukin-2 response, and the expansion of memory T cells have been documented in both human and murine aging. However, a direct relationship between the observed T cell hyporeactivity and clinical disease has not been established. Paradoxically, exaggerated inflammatory response has been linked to increased mortality from overwhelming sepsis [2,3], cardiovascular disease, and geriatric syndromes such as sarcopenia [4,5], frailty [6,7], as well as to all cause mortality [6]. There is a growing recognition that while inflammatory responses confer short term benefits, chronic low grade inflammation may actually contribute to the susceptibility and clinical manifestation of diseases in aging. However, the pathogenic basis behind these observations remains poorly understood. Furthermore, the source of the inflammatory mediators in aging is uncertain. Lastly, it is unclear if the inflammatory response observed in aging is secondary to disease or to the aging process.

Chemokines, or chemotactic cytokines, are a superfamily of at least forty small (8–10 kDa) proteins [8,9]. They are grouped together based on sequence homology and are classified according to the amino terminal cysteine motif into C, CC, CXC, and CX3C chemokines. At least 20 chemokine receptors have been cloned. Chemokines and their receptors are Janus molecules that participate in physiological responses and at the same time play a critical role in the pathogenesis of diverse diseases that are important in the elderly population, including sepsis [10], atherosclerosis [11,12], tumor rejection [11], diabetes [13,14] and rheumatoid arthritis [15]. Age-associated change in chemokine function may therefore have significant impact on both the normal and pathological inflammatory responses in the elderly. Importantly, the overlapping ligand-receptor specificity this system exhibits suggests a degree of redundancy. It is the coordinated interaction among chemokines and their receptors that define the *in vivo *multicomponent physiological and pathological responses [11]. Surprisingly, little is known about the effect of aging on the chemokine system.

CD8+ T cells, or cytotoxic T lymphocytes, are essential members of the immune system involved in tumor surveillance and in combating viral infections. A number of changes occur in CD8+ T cells in aging, including elevated percentage and number of memory CD8+ T cells, the accumulation of CD28-CD8+ T cells [16], and lost of Fas expression [17]. The coordinated migration of CD8+ T cells to peripheral tissues where they perform their effector function is a chemokine/chemokine receptor-dependent process. Thus, the absence of CCR7 impairs the magnitude of CD8+ T cell anti-viral response, with reduced number of virus-specific CD8+ cells in both lymphoid and nonlymphoid organs [18]. Similarly, low CCR4 and CXCR3 expression on CD8+ T cells has been linked to dampened anti-tumor response and poor survival in metastatic melanoma patients [19]. Paradoxically, CD8+ T cells are also involved in the pathogenesis of selected diseases prevalent in the elderly. For example, accumulation of CD8+ T cells in atherosclerotic plaques has been proposed as a mechanism contributing to the widespread apoptosis found within these lesions [20]. Aging associated changes in chemokine receptor expression may therefore alter important migratory and effector functions of CD8+ T cells. In this report, we investigated the role of aging in determining murine CD8+ T cell CC and CXC chemokine receptor gene expression.

## Results

### Aging and CD8+ T cell chemokine receptor gene expression

**Figure 1:**
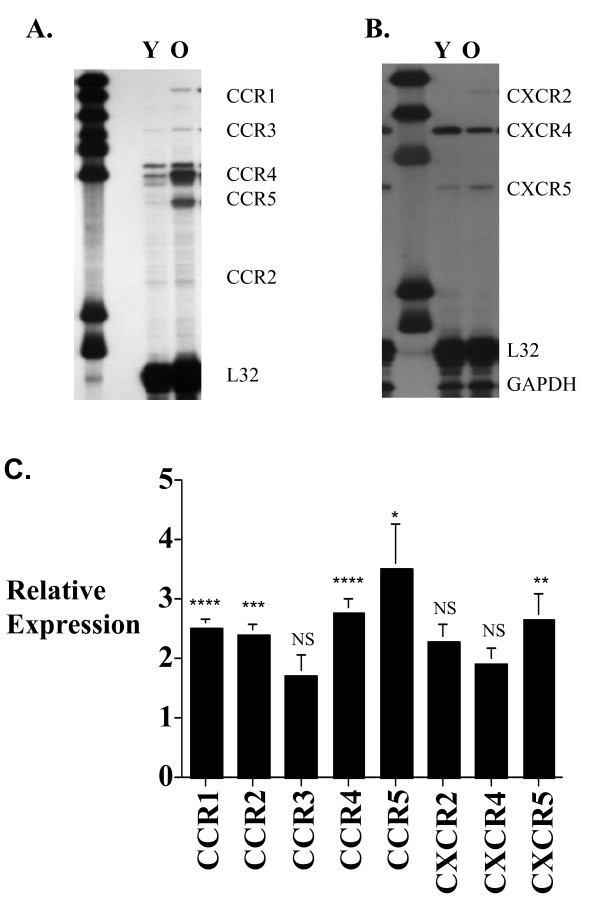
Murine CD8+ T cell chemokine receptor gene expression. Autoradiographs of representative ribonuclease protection assay (RPA) comparing young (Y) (3–4 months old) and old (O) (18–20 months old) C57BL/6 CD8 T cell CCR1–5 (A) and CXCR2, 4 and 5 (B) gene expression. Composite histogram (C) of 5 RPAs using pooled RNA from a total of 25 mice in each age group is shown. The quantitation was done by measuring the P^32^-radiation from identical size band areas on the RPA gel using a Phosphoimager. The results represent the mean ± SEM of the chemokine receptor gene expression level of old CD8+ T cells relative to young CD8+ T cells (arbitrarily defined as equal to 1). Gel loading is corrected with L32 expression. P value of ≤ 0.05 is considered statistically significant. * P ≤ 0.05, ** P ≤ 0.005, *** P ≤ 0.001, NS = not significant.

We compared the CC and CXC chemokine receptor gene expression of CD8+ T cells from young and old C57BL/6 mice using RPA probe sets that included CCR1–5, and CXCR2, 4 and 5 as before [21]. The results (Figure 1) indicated that freshly isolated old CD8+ T cells have significantly higher level of CCR1, CCR2, CCR4, CCR5 and CXCR5 gene expression than those from young CD8+ T cells. Smaller changes in CCR3, CXCR2 and CXCR4 gene expression were seen, but the results did not reach statistical significance.

### T cell receptor stimulation and T cell chemokine receptor expression

**Figure 2:**
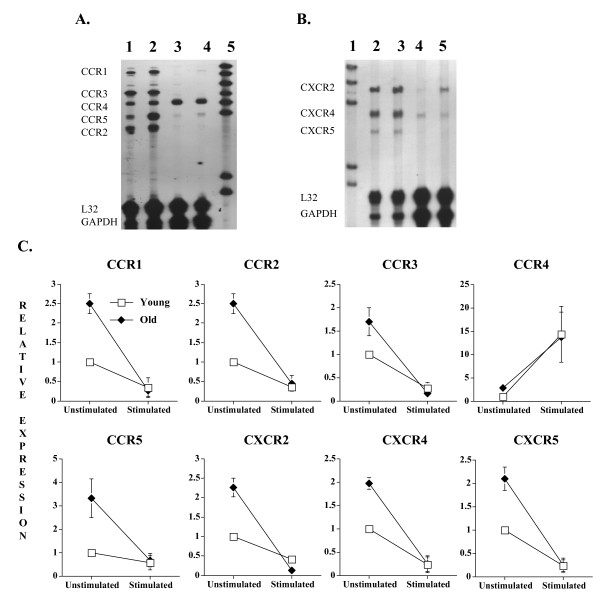
Murine CD8+ T cell chemokine receptor gene expression following T cell receptor/CD28 stimulation. Autoradiographs of representative ribonuclease protection assay (RPA) showing the effect of aging and anti-CD3/anti-CD28 monoclonal antibody (mAb) stimulation on murine CD8+ T cell CCR1–5 (A) and CXCR2, 4 and 5 (B) gene expression. Lane 1 in Figure 2A and Lane 2 in Figure 2B = Young (3–4 months) unstimulated cells; Lane 2 in Figure 2A and Lane 3 in Figure 2B = Old (18–20 months) unstimulated cells; Lane 3 in Figure 2A and Lane 4 in Figure 2B = Young cells after 72 hours of anti-CD3/anti-CD28 mAb stimulation; Lane 4 in Figure 2A and Lane 5 in Figure 2B = Old cells after 72 hours of anti-CD3/anti-CD28 mAb stimulation; Lane 5 in Figure 2A and Lane 1 in Figure 2B = unprotected probe set. Composite histogram (C) of 3 RPAs using pooled RNA from a total of 15 animals in each age group is shown. Gel loading is corrected with L32 expression.

Chemokine receptor expression profile is altered following the engagement of the TCR to the major histocompatibility complex (MHC) on antigen presenting cells. For example, receptors for constitutively expressed chemokines such as CXCR4 and CCR7 are down-regulated as naïve T cells are activated and differentiated into effector cells [22]. This in turn allows the CD8+ T cells to migrate to peripheral tissues to perform their effector function. Others have reported that specific T cell dysfunctions in aging can also be rescued by co-activating the TCR and T cell co-stimulation pathways [23-25]. We therefore determined the combined effect of T cell receptor (CD3)/costimulatory molecule (CD28) stimulation on young and old CD8+ T cell chemokine receptor expression profile (Figure 2). The results show that the CD8+ T cells CCR1, 2, 3, 5 and CXCR2, 4, 5 gene expression decreases after the CD3/CD28 activation. CCR4 is the only chemokine receptor that showed increased expression following anti-CD3/anti-CD28 mAb treatments, similar to what has been seen in CD4+ T cells [21]. Despite the age difference in basal chemokine receptor gene expression level, old CD8+ T cells exhibit similar robust CC and CXC chemokine receptor response to TCR/co-stimulatory activation as young CD8+ T cells.

### Caloric restriction and CD8+ T cell chemokine receptor expression

**Figure 3:**
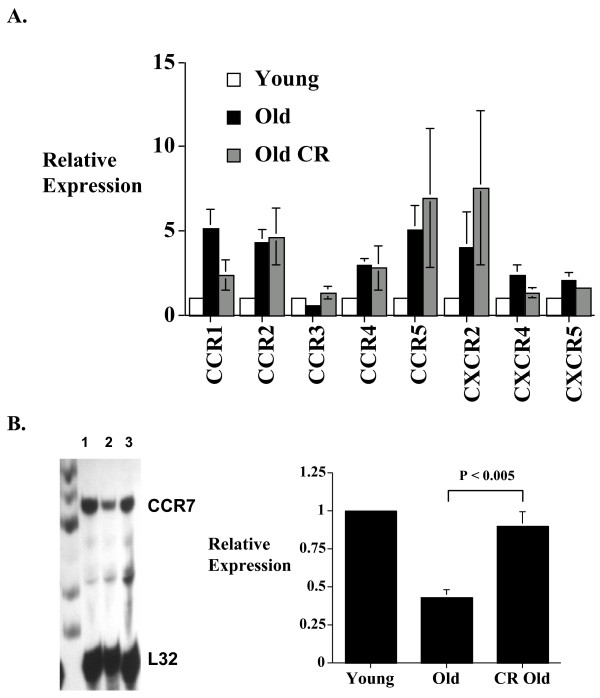
The effect of caloric restriction on CD8+ T cell chemokine receptor expression in aging. Ribonuclease protection assays (RPAs) were done using RNA from freshly isolated splenic CD8+ T cells from young (3–4 months), old (18–20 months), and caloric restricted old (18–20 months) mice in groups of 5 animals. Density of the bands was quantified using a phosphoimager. (A) Histogram showing the composite data of 4 experiments (total 20 animals in each condition). The results represent the mean ± SEM of the relative CD8+ T cell chemokine receptor gene expression level of old and old caloric restricted mice compared to those from the young cohort (arbitrarily defined as equal to 1). (B) CCR7 expression was also determined using a custom CCR7 RPA probe. The left panel is a representative autoradiograph (lane 1 = young CD8+ T cells; lane 2 = old CD8+ T cells; lane 3 = caloric restricted old CD8+ T cells). The right panel represents the composite data of 3 RPAs with a total of 15 animals in each group. Results are presented as mean ± SEM. CR = caloric restricted. Gel loading is corrected with L32 expression.

Caloric restriction has been shown to prolong life in mice and to restore many of the aging-associated defects in T cell immune functions [26]. In addition, caloric restriction may affect CD4+ T cell chemokine receptor gene expression in aging [21]. In the current study we did not see any significant effect of caloric restriction on CD8+ T cell chemokine receptor expression compared to ad lib fed mice (Figure 3A). Although the CD8+ T cell CCR1, CCR3 and CXCR4 expression in caloric restricted mice approximates that seen in the young mice, the results did not reach statistical significance. We also examined the effect of caloric restriction on CCR7 expression using a custom RPA probe set as before [21]. The results show reduced CCR7 expression in old CD8+ T cells, similar to that reported in CD4+ T cells in aging [21,27]. Interestingly, caloric restriction selectively prevents the loss of CCR7 expression in CD8+ T cells (Figure 3B).

### Chemokine receptor protein levels in CD8+ T cells in aging

**Figure 4:**
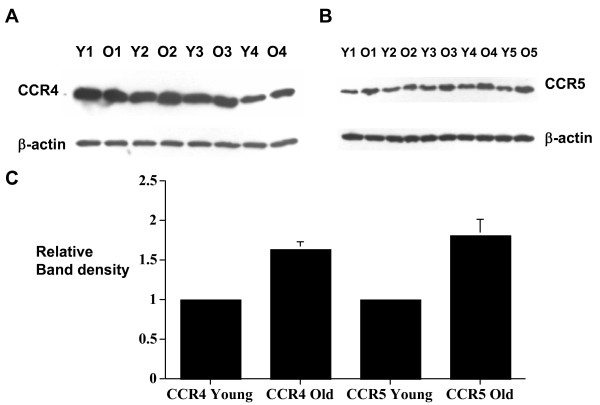
Western blot analyses of CCR4 and CCR5 protein level in aged CD8+ T cells. Intracellular proteins were isolated from groups of 4–6 young and old C57Bl/6 mice. (A) Western blot analysis of CCR4 using proteins from 4 groups of young (Y1–Y4) and old (O1–O4) CD8+ T cells (total 20 young and 20 old mice). (B) Western blot analysis of CCR5 on CR8+ T cells isolated from 5 groups of young (Y1–Y5) and Old (O1–O5) mice (total 25 young and 25 old mice). (C) Histogram showing the composite results of the relative CCR4 and CCR5 protein level of old CD8+ T cells compared to young CD8+ T cells. Pairwise comparison was done for each individual sample preparation (Y1 versus O1, Y2 versus O2 etc., with the young group arbitrarily assigned the value of 1). The results are corrected for gel loading using β-actin as controls. The results are presented as mean ± SD.

Since most chemokine receptors remain in the intracellular compartment and cell surface chemokine receptors constantly go through clathrin-mediated internalization and recycling, flow cytometric analysis of chemokine receptor cell surface expression may not correlate with changes in chemokine receptor gene expression. We therefore determined the chemokine receptor protein level by Western blot analysis using total cell lysate. Western blot analyses were done using anti-CCR4 and anti-CCR5 antibodies as before [21]. The results showed that the detected increase CCR4 and CCR5 gene expression in CD8+ T cells in aging correlates with increase CCR4 and CCR5 protein levels in aging (Figure 4).

### CD8+ T cell MIP-1α chemotaxis response in aging

**Figure 5:**
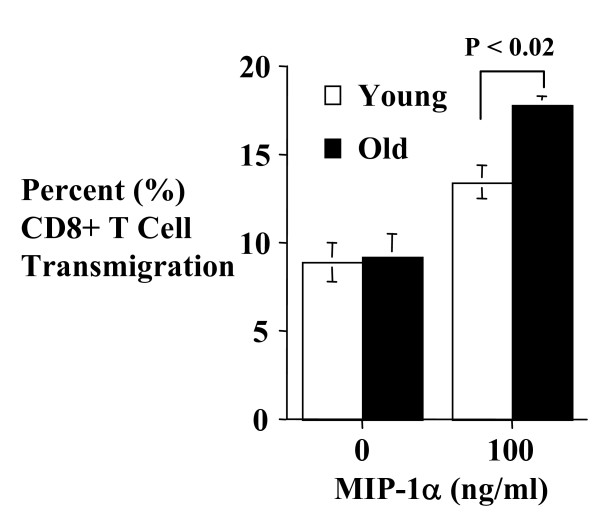
Effect of aging on murine CD8+ T cell chemotaxis response to MIP-1α. The indicated concentrations of the chemokine were placed in the lower chamber of a Costar Transwell system. Equal number of freshly isolated young (3–4 months) or old (18–20 months) CD8+ T cells was then placed in the upper chamber of the insert, and the number of cells in the upper and lower chambers counted 5 hours later using a Coulter counter. The results are expressed as percent transmigration and represent the mean ± SD of triplicate determinations. P ≤ 0.05 is considered significant. The results are representative of 3 independent experiments.

To determine if increased chemokine receptor gene expression correlates to increase CD8+ T cell chemotaxis function, we compared the *in vitro *migration response of young and old CD8+ T cells to MIP-1α (Figure 5), a ligand for CCR1 and CCR5. The results showed increased migration of old CD8+ T cells to the chemokine, confirming functional significance of the increased CCR1 and 5 expressions in old CD8+ T cells.

## Conclusion

Aging is associated with a myriad of changes in immune functions. Despite the widely held notion that aging causes impaired immune and inflammatory responses, there is now considerable evidence that not all aspects of immune functions are adversely affected in the elderly. Little is known about the effect of aging on T cell chemokine response. We have previously shown that murine CD4+ T cells expressed a higher level of CCR1, 2, 4, 5, 6 and 8 and CXCR2–5, and lower level of CCR7 and 9 [21]. To the best of our knowledge, the current study is the first systematic report of the effect of aging on CD8+ T cell chemokine receptor gene expression. Our results demonstrated age-related increase in CD8+ T cell CCR 1, 2, 4, 5 and CXCR5, and reduced CCR7 gene expression. These results are similar to that we reported in CD4+ T cells, suggesting that similar mechanisms are responsible for the observed aging-dependent differences in both cell types. We further showed that increase CCR4 and CCR5 gene expression in aging correlates to increase CCR4 and CCR5 protein levels.

The downregulation of selected chemokine receptor following TCR activation is consistent with what has been reported in the literature [21,27-31]. One group of investigators showed that memory/effector T cells undergo a further but transient switch in chemokine receptor expression following TCR stimulation, including down regulation of CCR1, 2, 3, 5, 6 and CXCR3, and upregulation of CCR4 [22]. In the current study, we also found that CCR4 expression increases following anti-CD3/CD28 stimulation. Despite the elevated basal level of chemokine receptor genes, old CD8+ T cells showed a robust response to T cell receptor/co-stimulatory activation to a similar degree seen in their younger cohort.

Memory and naïve T cells tend to express a different combination of chemokine receptors. Aging is also known to be associated with reduced CD8 T cell clonal (antigen receptor) diversity. The reason for the observed chemokine receptor gene expression changes may therefore be in part explained by the bias toward memory T cells in aging. However, although we did not specifically investigate the naïve and memory CD8+ T cell subsets, this seems unlikely to be the sole explanation as we have previously shown that aging has similar effect on naïve and memory CD4+ T cells chemokine receptor gene expression [32]. Furthermore, although old caloric restricted mice have fewer memory T cells [26], our results showed that they have similar T cell chemokine receptor expression profile as old mice receiving ad lib feeding (except CCR7). Finally, our data showed that although the memory T cell-associated chemokine receptors (e.g. CCR4 and CCR5) are upregulated in aging, we also detected a small increase in naïve T cell associated chemokine receptor CXCR4 that can not be due to the reduced naïve T cell population in aging.

Restricting caloric intake has been shown to extend life span in invertebrates (e.g. C. elegans, Drosophilla), short-lived vertebrates (e.g zebra fish) and rodents [reviewed in [33]]. In addition, caloric restriction is a key environmental manipulation that has been shown to modulate T cell function in aging, including maintaining naïve T cell population, T cell proliferation capacity and death responses. Surprisingly, our results showed that caloric restriction has relatively little effect on CD8+ T cell chemokine receptor gene expression, suggesting a different mechanism of control. As stated above, the only exception is CD8+ T cell CCR7 expression. Similar to what we and others have reported in CD4+ T cells [21,27], we found that CCR7 expression on freshly isolated CD8+ T cells decreases with aging in ad lib fed mice. This is also consistent with a recent report showing decreased CCR7 expression in the respiratory syncytial virus-specific CD8+ memory T cell responses in elderly persons [33]. However, our results do not exclude the obvious possibility that increased in memory T cells in aging may be responsible for the observed CCR7 results. Interestingly, CCR7 expression in caloric restricted mice is similar to the level seen in young CD8+ T cells. The reason for the selected CCR7 response to caloric restriction is unknown. Recent works suggest that CCR7 may be under the control of epigenetic factors [34], and we are currently exploring this as a potential mechanism controlling chemokine receptor expression in aging. Of interest, CCR7 has been reported to play an important role in protecting CD8+ T cells from apoptosis [35]. It is therefore tempting to postulate that the low CCR7 expression in aging CD8+ T cells may contribute to their resistant to undergo apoptosis that some investigators have reported [36,37]. A role of CCR7 in CD8+ T cell in lymphoid migration has also been firmly established [38]. The observed age-associated reduction in CCR7 expression may therefore help explain the observed impaired lymphocyte lymphoid homing in aging [39-42]. Lastly, recent investigation showed that CCR7-/- mice have prolonged allograft survival accompanied by a delay in the cellular infiltration of the allograft [43]. Reduced T cell CCR7 expression in aging provides a potential novel mechanism contributing to the observed reduced allograft rejection and improved organ transplantation in the elderly [44,45].

The observed aging changes in CD8+ T cell chemokine receptor gene expression may also have an impact on other aging related conditions. For example, CCR1-/- mice have improved survival in experimental sepsis [10] and the age-related increase in T cell CCR1 may contribute to the observed increased mortality in septicemia in aging [46,47]. Mice lacking CCR5 unexpectedly were found to have enhanced delayed-type hypersensitivity and increased humoral response to T cell-dependent antigenic challenge [48]. The immunosuppressive effect of CCR5 is also seen in a recent report showing that CCR5 plays a role in downregulating donor alloreactive CD8+ T-cell expansion in allogeneic bone marrow transplants [49]. Thus, the increased CCR5 expression on aged T cells may also contribute to the observed reduced allograft rejection and increased susceptibility to infection in aging.

Aging can be defined as a process where an individual organism progressively becomes increasingly susceptible to disease (e.g. infections and cancer) and mortality. The mechanisms for aging are not well defined, with genetic, epigenetic and environmental factors implicated in its pathogenesis. Our study provides the first systematic analysis of murine CD8+ T cell chemokine receptor gene expression in aging. The observed selected CD8+ T cell CC and CXC chemokine receptor overexpression in aging may predispose the elderly to chemokine-dependent diseases such as sepsis and cardiovascular disease. Conversely, impaired CCR7 expression may provide a mechanism for impaired T cell lymphoid homing anti-viral responses, and improved allograft survival in the elderly population.

## Methods

### Mice

Young (3–4 months), old (18–20 months) and old caloric-restricted (18–20 months) C57BL/6 mice were obtained from the National Institute on Aging (NIA) Aged Rodent Colonies through Harlan Sprague Dawley (Indianapolis, IN). The caloric restriction protocol is published in the NIA website. Briefly, caloric restriction is initiated at 14 weeks of age at 10% restriction, then 25% and 40% restriction at 15 and 16 weeks respectively [21]. All mice were maintained in a pathogen-free environment provided by the Unit for Laboratory Animal Medicine at the University of Michigan (Ann Arbor, MI) until they were used. Careful inspection of the animal was done at the time of sacrifice to exclude aged animals with cancer or lymphoma. All the experimental research in the current study have been approved by the University of Michigan University Committee on Use and Care of Animals (UCUCA).

### CD8+ T cell isolation

Splenic CD8+ T cells were negatively selected using a combination of CD4 (L3T4), CD45R (B220), CD49b (DX5), CD11b (Mac-1) and Ter-119 microbeads (Miltenyi Biotec, Auburn, CA) as before [21,50]. Alternately, in some experiments the cells were isolated by positive selection using the CD8a (Ly-2) microbeads. Purity of the cells isolated using either method was confirmed by flow cytometric analyses, and was consistently >96%.

### T cell culture and T cell receptor (TCR) stimulation

All the monoclonal antibodies (mAbs) were obtained from BD PharMingen (San Diego, CA) unless otherwise stated. Combined anti-CD3 and anti-CD28 mAbs were used to provide maximum TCR/co-stimulation to the CD8+ T cells [21]. Briefly, anti-CD3e (2.5 μg/ml final concentration) was diluted in PBS and immobilized to the individual wells of 6-well flat bottom tissue culture plates (Corning Glass Works, Corning, NY) in a final volume of 6 ml for overnight. The plates were then washed with PBS twice. 1 × 10^6 ^purified CD8+ T cells were then cultured in 6 ml media containing RPMI 1640 medium supplemented with 10% FBS, 2-ME and anti-CD28 (2.25 μg/ml final concentration) in a humidified atmosphere at 5% CO_2 _at 37°C for 72 hours. RNAs from unstimulated and anti-CD3/anti-CD28 stimulated cells were isolated by TRIzol^®^LS reagent (Life Technologies, Grand Island, NY), and a second cleanup step performed using the Qiagen RNeasy Total RNA isolation kit (Qiagen, Valencia, CA). Intracellular proteins were isolated from the phenol-ethanol supernate with isopropyl alcohol after precipitation with ethanol, as per standard protocol.

### RNase protection assays (RPAs)

Chemokine receptor gene expression was quantified by RPAs as before [21,50]. Briefly, pooled RNAs from equal number of purified CD8+ T cells from young and old mice in groups of 4–6 animals were used for each experiment to minimize individual variability. The probes were synthesized by modification of the manufacturer's protocol. Briefly, GACU nucleotide pool and [α-^32^P]UTP, RNasin, T7 RNA polymerase were added to the multi-probe template set mCR-5 (CCR1–5), mCR-6 (CXCR2, 4, and 5) or a custom probe set containing CCR7 [21] (all from BD PharMingen, San Diego, CA) and placed on heat block at 37° for 1 hour. The reaction was terminated by adding DNase and incubated at 37°C on a heat block for 30 minutes. Appropriate volumes of EDTA, Tris-saturated phenol, chloroform:isoamyl alcohol (CIAA, 50:1) and yeast tRNA were then added to the mixture, as suggested by the manufacturer. The aqueous layer was extracted by CIAA, then pelleted by adding a 1:5, 4 M ammonium acetate and ice-cold 100% ethanol mixture. 5 μg of total RNA from each T cell sample was used for hybridization. The protected probes were then fractionated by electrophoresis through a 5% acrylamide gel, exposed to a phosphor screen and quantified by a Phosphorimager using Image Quant software (Molecular Dynamics). The signals quantified were in the linear range.

### *In vitro *chemotaxis assays

Dual chamber chemotaxis assays were performed to compare the MIP-1α (CCL3; ligand for CCR1 and 5) (PeproTech, Rocky Hill, NJ) response of CD8+ T cells from young and old animals. Briefly, freshly isolated 4 × 10^5 ^T cells in 100 μl RPMI 1640 medium supplemented with 0.5% BSA were placed in Transwell Clear culture inserts with 5 μm pores (Corning-Costar, Cambridge, MA). The inserts were then placed in 24-well tissue culture plate (Corning-Costar) containing 600 μl of the indicated concentrations of murine MIP-1α in RPMI 1640 medium supplemented with 0.5% BSA for 5 hours in a humidified incubator at 37°C. Cells from the top and bottom chambers were then harvested and counted with a Beckman Coulter counter.

### Western blots

Proteins from young and old CD8+ T cells were resolved on 10% SDS-polyacrylamide gels and transferred to Nitrocellulose-1 membrane (Life Technologies, Inc., Gaithersburg, MD). The membrane was blocked in PBS containing 5% nonfat dry milk, and 0.05% Tween 20 and subsequently incubated with anti-mouse CCR4 or CCR5 antibodies (BD PharMingen) followed by HRP-conjugated anti-rabbit and anti-rat IgG F(ab')2 (Amersham Life Science). Detection was performed using the ECL system (Amersham). The membranes were then stripped and re-probed with anti-mouse-β-actin antibodies (Sigma, MO) to confirm equal protein loading.

### Statistical analyses

Data were analyzed using analysis of variance (ANOVA) or Student's *t*-test, with Bonferroni corrections for multiple comparisons where appropriate. Statistical significance was set at P < 0.05.

## Competing interests

The author(s) declare that they have no competing interests.

## Authors' contributions

RY had overall responsibility for the experimental design, statistical analysis, and wrote the manuscript.

RM carried out the experiments in the manuscript.

AG consulted on the results and manuscript preparation.

MH consulted on the results and manuscript preparation.

All the authors have read and approved the final manuscript.
